# Simultaneous Determination of Three Bioactive Constituents from *Bletilla striata* by UPLC-MS/MS and Application of the Technique to Pharmacokinetic Analyses

**DOI:** 10.1155/2019/8942512

**Published:** 2019-10-21

**Authors:** Hao Chen, Lin Zheng, Chao-Ye Mei, Zi-Peng Gong, Yong-Jun Li, Si-Ying Chen, Yan-Yu Lan, Yong-Lin Wang, Ai-Min Wang, Yue-Ting Li, Yong Huang

**Affiliations:** ^1^State Key Laboratory of Functions and Applications of Medicinal Plants, Guizhou Provincial Key Laboratory of Pharmaceutics, Guizhou Medical University, Guiyang 550004, China; ^2^School of Pharmacy, Guizhou Medical University, Guiyang 550004, China; ^3^Guizhou Provincial Engineering Research Center for the Development and Application of Ethnic Medicine and TCM, Guizhou Medical University, Guiyang 550004, China

## Abstract

*Bletilla striata* has been widely used as a valuable hemostatic agent for thousands of years due to the high levels of bioactive constituents it contains. Here, we used a sensitive ultrahigh-performance liquid chromatography-tandem mass spectroscopy (UPLC-MS/MS) method for the simultaneous determination of three major active ingredients of the *B. striata* extract, namely, *α*-isobutylmalic acid, gymnoside I, and militarine in rat plasma. The three major active ingredients were determined using the multiple reaction monitoring (MRM) mode at *m/z* 189 ⟶ 129 for *α*-isobutylmalic acid, *m/z* 457.2 ⟶ 285.1 for gymnoside I, *m/z* 725.3 ⟶ 457.2 for militarine, and *m/z* 417.0 ⟶ 267.0 for the IS puerarin. All calibration curves showed good linearity (*R*^2^ ≥ 0.999) over the concentration range with the lower limit of quantification between 0.015 and 0.029 *μ*g/mL. The relative standard deviations of intraday and interday measurements were less than 15%, and the method was accurate within 93.3–100.4%. The extraction recovery was 92.65–100.98%, and no matrix effect was observed. The results indicated that after oral administration of *B. striata* in rats, the *T*_max_ of *α*-isobutylmalic acid was significantly longer than that of gymnoside I and militarine and the mean residence time and area under the curve of *α*-isobutylmalic acid and gymnoside I in rats were significantly higher than those of militarine. Moreover, the blood concentration-time curve of *α*-isobutylmalic acid showed double peaks, suggesting that *α*-isobutylmalic acid could exhibit the phenomenon of enterohepatic circulation or metabolic conversion. We also explored some of the pharmacokinetic characteristics of three ingredients from *B. striata* extract *in vivo*, and the data obtained may provide a basis for the further investigation of *B. striata*.

## 1. Introduction

*Bletilla striata* (Thunb.) Reichb. f. (Orchidaceae) is a medicinal plant, the dry tubers of which have a hemostatic effect and are prescribed by the Chinese Pharmacopoeia as a medicinal part [1]. It is widely used in traditional Chinese herbal medicines for thousands of years in China and first recorded in Shennong's Materia Medica [2]. It is bitter, sweet, and puckery in flavor and slightly cool and astringent in nature, and it acts on the lung, liver, and stomach meridians [1, 2]. The dried tubers of *B. striata* can effectively inhibit leakage of blood and stop bleeding, disperse swelling, and promote tissue regeneration [3]. Thus, it has been used as a valuable hemostatic agent for the treatment of hemoptysis, vomiting blood, traumatic bleeding, and ulcerative carbuncles [4, 5]. Furthermore, it is not only used in the treatment of sores, chapped skin, and swelling [6, 7] but also has antibacterial [8, 9] and antitumor effects [10–12]. After years of research, more than 90 compounds have been isolated from *B. striata* tubers, including benzyl compounds, phenanthrene, glycosides, anthocyanins, triterpenes, and steroids [10, 13–15]. Moreover, our previous pharmacological work has confirmed that *B. striata* extract shows a hemostatic effect by promoting platelet aggregation [16, 17].

However, most of these reports only focus on the pharmacological effects and chemical composition of *B. striata* extract, and pharmacokinetic studies on *B. striata* extract have not been reported. Adequate pharmacokinetic data for traditional medicines are critical to ensure safe and proper application. To study the pharmacokinetic properties, the primary active ingredients are typically selected to characterize the pharmacokinetic profile of these drugs. The study will provide a method to explore the pharmacokinetic characteristics of the main representative components of *B. striata* clinical applications. According to serum medicinal chemistry, the pharmacological activity of bioactive ingredients depends largely on whether they are absorbed into the blood and maintain a certain blood concentration [18]. In our previous study, we found that *α*-isobutylmalic acid, gymnoside I, and militarine are relatively high in *B. striata* extract [19], and after oral *B. striata* extract in rats, three components were detected in the serum [20]. Recent research has shown that militarine can not only be metabolized to *α*-isobutylmalic acid and gymnoside I in zebrafish but also that it is the most abundant active component in *B. striata* [21–23]. Furthermore, militarine can improve intelligence, learning ability, and memory and can also prevent senile dementia and plays a protective role against aging-related neural defects [24, 25]. At the same time, studies have shown that *α*-isobutylmalic acid and gymnoside I may be potential pharmacological components [26, 27]. Therefore, we selected *α*-isobutylmalic acid, gymnoside I, and militarine as indicative active components of *B. striata* extract to study the pharmacokinetics and absolute bioavailability of these three compounds in rats.

Here, we report the development and validation of a novel, sensitive, and selective UPLC-MS/MS method for the simultaneous determination of *α*-isobutylmalic acid, gymnoside I, and militarine concentrations and its application to investigate their pharmacokinetics and absolute bioavailability in the rat's plasma after intravenous injection and oral administration of *B. striata* extract.

## 2. Materials and Methods

### 2.1. Materials and Reagents

**Figure 1:**
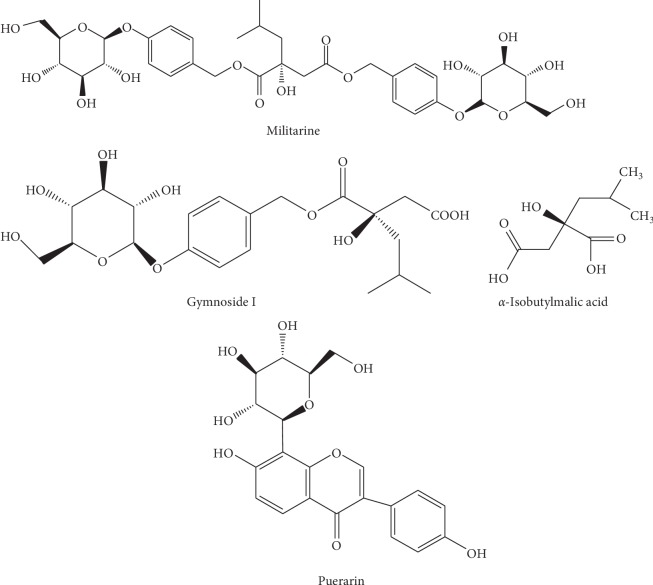
Chemical structures of *α*-isobutylmalic acid, gymnoside I militarine, and puerarin (internal standard).

The standards of puerarin (purity: 95.4%) were obtained from the National Institutes for Food and Drug Control (Beijing, China). *α*-Isobutylmalic acid, gymnoside I (purity ≥ 95%), and militarine (purity ≥ 98%) were isolated from *B. striata* in our laboratory. The structure and purity of these compounds were confirmed using IR, ^1^H nuclear magnetic resonance (NMR), MS, and HPLC-UV. The structures of the three components and puerarin are shown in Figure 1. HPLC-grade acetonitrile and methanol were purchased from Merck Co. (Darmstadt, Germany). HPLC-grade formic acid was supplied by Dikma (Richmond Hill, NY, USA). Distilled water was obtained from Watsons Group Co. Ltd. (Hong Kong, PR China). All other chemicals were of analytical grade.

The extraction of *B. striata* was conducted using a method described by Zhao et al. [16]. The dried medicinal *B. striata* (10 kg) was powdered and extracted 3 times (2 h each time) with 95% ethanol (1 : 4 m/v). The decoction was combined and concentrated under reduced pressure to 10 L. The residue was directly subjected to D101 macroporous resin eluted with 80% aqueous ethanol. The elution liquid was finally concentrated under reduced pressure, and the concentrates were dried to powder by microwave vacuum. The *α*-isobutylmalic acid, gymnoside I, and militarine contents of the *B. striata* extracts were determined under the same chromatography conditions as described in the following sections and found to be 2.36%, 1.07%, and 26.37%, respectively.

### 2.2. Animals

Sprague-Dawley rats (weighing 230 ± 20 g) were provided by Chongqing Tengxin Biotechnology Co., Ltd. (Chongqing, China, certificate No. SCXK (Yu) 2015-0001). All studies were approved by the Animal Ethics Committee at Guizhou Medical University and conducted in accordance with the guidelines of the Committee on the Care and Use of Laboratory Animals in China.

### 2.3. Instrumentation and Analytical Conditions of UPLC-MS/MS

An ACQUITY™ UPLC system (Waters Corp., Milford, MA, USA) with a conditioned autosampler and the ACQUITY™ UPLC BEH C_18_ analytical column (50 mm × 2.1 mm, internal diameter, 1.7 *μ*m; Waters Corp., Milford, MA, USA) were used, to which a 6 mm precolumn filter unit was added. Analysis was carried out with an elution gradient of (A) acetonitrile and (B) water (both containing 0.1% formic acid) at a flow rate of 0.35 mL/min as follows: 0–0.5 min (10% A), 0.5–1.0 min (10–30% A), 1.0–2.0 min (30–35% A), 2.0–3.0 min (35–38% A), 3.0–4.0 min (38–90% A), and 4.0–5.0 min (90–10% A). The column and autosampler were maintained at 45°C and 15°C, respectively. The injection volume was 2 *μ*L.

Mass spectrometric detection was performed using a Waters ACQUITY™ TQD triple quadrupole tandem mass spectrometer (Waters Corp., Manchester, UK) with an ESI interface in the positive ionization mode. The MS conditions were as follows: capillary voltage, 3.0 kV; desolvation gas flow, 650 L/h of nitrogen; and cone gas flow, 50 L/h of nitrogen. The collision gas (Ar) flow for MS/MS was maintained at 0.16 mL/min for collision-induced dissociation (CID). The source and desolvation gas temperatures were 120°C and 350°C, respectively. The multiple reaction monitoring (MRM) mode was used for quantification. The optimal MRM mode parameters of the analytes and internal standard (IS) are given in Table 1. All data were acquired using Masslynx™ V 4.1 Software and processed using Quanlyna™ V4.1 (Waters Corp., Millford, MA, USA).

### 2.4. Standard Solution, Calibration Standards, and Quality Control (QC) Samples

Stock solutions of puerarin (IS), *α*-isobutylmalic acid, gymnoside I, and militarine were prepared by dissolving the appropriate amounts of each standard substance in 10 mL of methanol to yield concentrations of 2.90 mg/mL, 1.50 mg/mL, 1.10 mg/mL, and 0.405 mg/mL, respectively. Working solutions for calibration were prepared at concentrations of 0.015, 0.029, 0.145, 0.454, 1.362, 4.086, 12.259, 36.778, and 110.333 *μ*g/mL for *α*-isobutylmalic acid, 0.011, 0.022, 0.11, 0.55, 2.75, 13.75, and 27.50 *μ*g/mL for gymnoside I, and 0.015, 0.098, 2.642, 7.926, 23.778, and 99.333 *μ*g/mL for militarine. Quality control (QC) samples were prepared, containing *α*-isobutylmalic acid (0.029, 4.09, and 36.78 *μ*g/mL), gymnoside I (0.02, 2.75, and 13.75 *μ*g/mL), militarine (0.015, 2.64, and 23.78 *μ*g/mL), and gymnoside I (0.6, 19.2, and 57.6 *μ*g/mL) using the same method as for the calibration samples. All the stocks and working solutions were stored at 4°C and brought to room temperature before use.

### 2.5. Sample Preparation

Ten microliters of the IS solution (puerarin, 10 *μ*g/mL in methanol) and 50 *μ*L of 1% formic acid were added to 100 *μ*L of rat plasma. Proteins were precipitated by addition of 400 *μ*L of methanol, vortexing for 1 min, sonication for 5 min, followed by centrifugation at 13225 ×*g* for 10 min at 4°C, collecting the supernatant in a centrifuge tube, and drying at 37°C N_2_. Subsequently, 300 *μ*L of the initial mobile phase was dissolved, sonicated for 5 min, and centrifuged at 5°C and 13225 ×*g* for 5 min, and the supernatant was used for analysis by UPLC-MS/MS.

### 2.6. Method Validation

Method validation for the quantification of these *B. striata* compounds in rat plasma was performed by following the US Food and Drug Administration Bio-analytical Method Validation Guide with the following parameters evaluated: selectivity specificity, matrix effect, linearity, accuracy, precision, recovery, and stability.

#### 2.6.1. Specificity and Selectivity

Specificity and selectivity of the method were evaluated by analyzing blank rat plasma, blank rat plasma spiked with *α*-isobutylmalic acid, gymnoside I, militarine, and IS, and rat plasma collected at 0.5 h after oral and intravenous administration of *B. striata* extract spiked with IS.

#### 2.6.2. Linearity and Lower Limit of Quantification

To investigate linearity and lower limit of quantification (LLOQ), the stock solution of each analyte was diluted with methanol to make a series of working solutions. The calibration samples of each analyte were prepared independently by adding a series of different concentrations of working solution (50 *μ*L), IS solution (10 *μ*L, 10 *μ*g/mL), formic acid (50 *μ*L, 1%), and 400 *μ*L methanol to blank rat plasma (100 *μ*L) to determine linearity and LLOQ. The LLOQ was defined as the lowest concentration on the calibration curve that could fulfill the analytical requirement of S/N ≥ 10 with an acceptable accuracy and precision (within ±20%).

#### 2.6.3. Precision and Accuracy

Precision was evaluated using relative standard deviation (RSD), and accuracy was determined via analytical recovery. The accuracy and the precision of the assay for intraday and interday determinations were evaluated by the analysis of three QC samples (*n* = 5) on the same day and on three consecutive validation days.

#### 2.6.4. Extraction Recovery and Matrix Effect

The extraction recoveries of three analytes were determined by comparing the peak areas of the low-, middle-, and high-QC samples prespiked in blank plasma with those postspiked in blank plasma (*n* = 5). The matrix effect was determined by comparing the peak areas of the QC samples prespiked in blank plasma with those in the initial mobile phase (*n* = 5).

#### 2.6.5. Stability

The stability of each compound in rat plasma was evaluated using mixtures containing low, medium, or high concentrations of the QC sample (*n* = 5 for each concentration level). The stability of each compound was tested under three conditions: room temperature for 6 h, three freeze (−20°C)-thaw (room temperature) cycles, and storage at 4°C for 12 h.

### 2.7. Pharmacokinetic Study

The twelve Sprague-Dawley rats were housed under a controlled temperature regimen (22 ± 1°C) and relative humidity of 50–70%. The animals were maintained on a 12 h light/12 h dark cycle and had free access to food and water. They were fasted for 12 h before each experiment but allowed to drink freely during this time. Before the experiment, the *B. striata* extracts' intravenous and oral dosing solutions were prepared in physiological saline at concentrations of 1.03 g/mL and 2.78 g/mL, respectively. The 12 rats were randomly divided into two groups with 6 rats in each group. Group 1 underwent oral administration (22.2 g/kg), and group 2 underwent intravenous administration (1.64 g/kg). Blood samples (0.3 mL) were collected from the external right jugular veins of the rats into heparinized tubes at the designated time points (0.033, 0.083, 0.167, 0.33, 0.5, 1, 1.5, 2, 10, 24, 36, and 48 h for group 1 and 0.033, 0.083, 0.167, 0.25, 0.33, 0.5, 0.75, 1, 1.6, and 2 h for group 2). The blood samples were immediately centrifuged at 3000 ×*g* for 5 min. The plasma was removed and frozen at −20°C until further analysis.

### 2.8. Statistical Analysis

Data are represented as mean± standard deviation. To determine the pharmacokinetic parameters, the concentration-time data were analyzed by DAS 2.0 software (Mathematical Pharmacology Professional Committee of China, Shanghai, China). Statistical analysis was performed using the statistical software package Statistical Product and Service Solutions (SPSS 11.5, USA). The absolute bioavailability was calculated using the following equation: F (%) = [AUC0 − *t* (oral) × dose (i.v.)]/[AUC0 − *t* (i.v.) × dose (oral)]) × 100.

## 3. Results and Discussion

### 3.1. Method Validation

#### 3.1.1. Specificity and Selectivity

**Figure 2:**
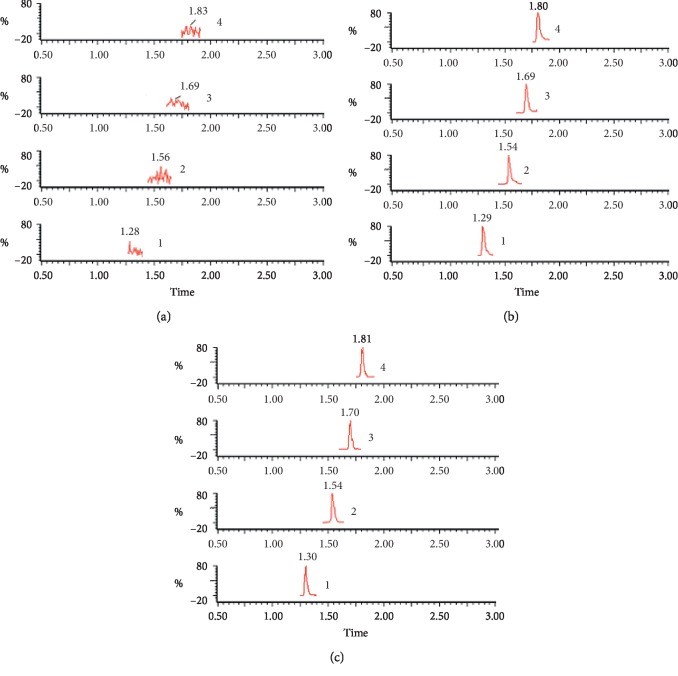
Chromatograms of the three analytes and (internal standard, IS) in rat plasma. Blank plasma sample (a); blank rat plasma sample spiked with three analytes (*α*-isobutylmalic acid, gymnoside I and militarine) and IS (quercetin) (b); rat plasma sample obtained 0.5 h after oral administration and intravenous administration of *B. striata* extract (c). (1) Puerarin (IS), (2) *α*-isobutylmalic acid, (3) gymnoside I, (4) militarine.

The specificity of the method was determined by comparing the chromatograms of blank (unspiked) plasma with the corresponding spiked plasma. Typical chromatograms of blank plasma (A), spiked plasma (B), and plasma from a pharmacokinetic study (C) are shown in Figure 2. The retention times for *α*-isobutylmalic acid, gymnoside I, militarine, and puerarin (IS) were 1.54, 1.70, 1.81, and 1.30 min, respectively. There was no endogenous interference in the MRM mode for any of the analytes.

#### 3.1.2. Linearity and Lower Limit of Quantification

Typical equations for calibration curves and LLOQ of the three analytes are shown in Table 2. All the three analytes exhibited good linearity with correlation coefficients within the range of 0.9991–0.9994.

#### 3.1.3. Precision and Accuracy

As shown in Table 3, the results for intraday and interday precision and accuracy for the three compounds indicated that the intraday and interday RSDs were all less than 10.4% and 5.0%, respectively, while the corresponding accuracy ranged from 93.3% to 100.4%. These data suggest that both precision and accuracy achieved with this method are acceptable.

#### 3.1.4. Extraction Recovery and Matrix Effect

The matrix effects can be regarded; all ratios (A/B × 100) % were between 93.21% and 101.74%, showing that there was no significant interference from endogenous substances. The extraction recoveries were in the range 92.65% to 100.98%. These results showed that all values were within the acceptable ranges (Table 4).

#### 3.1.5. Stability

The three analytes were stable under all testing conditions, including room temperature for 6 h, three freeze (−20°C)-thaw (room temperature) cycles, and storage at 4°C for 12 h. The results for all conditions are summarized in Table 5.

### 3.2. Pharmacokinetics

**Figure 3:**
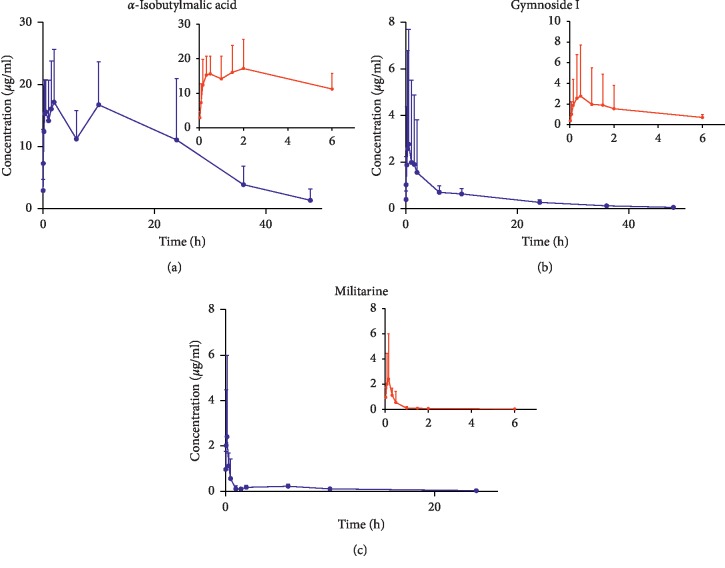
Mean plasma concentration-time curves of *α*-isobutylmalic acid, gymnoside I, and militarine after oral administration of *B. striata* extract to rats (*n* = 6).

**Figure 4:**
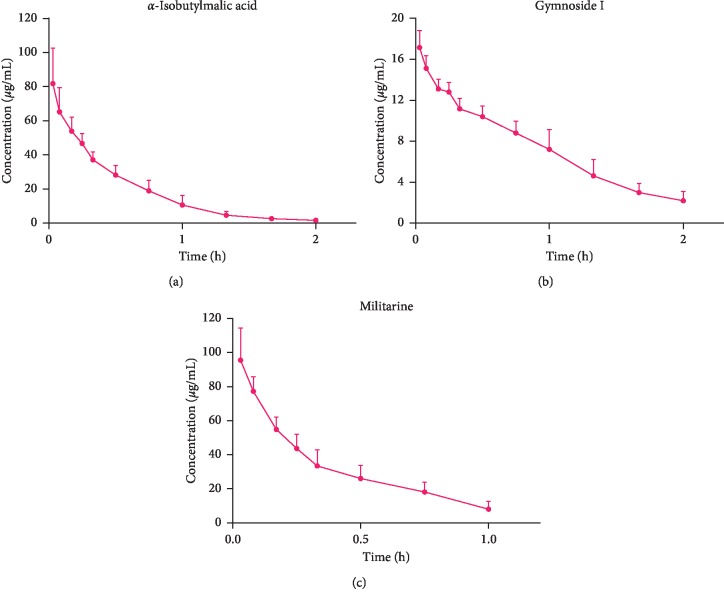
Mean plasma concentration-time curves of *α*-isobutylmalic acid, gymnoside I, and militarine after intravenous administration of *B. striata* extract to rats (*n* = 6).

The developed and validated UPLC-MS/MS method was successfully applied for the determination of *α*-isobutylmalic acid, gymnoside I, and militarine pharmacokinetics. The drug concentration-time curves after the intravenous injection of 1.64 g/kg *B. striata* extract and oral administration of 22.2 g/kg *B. striata* extract are shown in Figures 3 and 4. The pharmacokinetic parameters are given in Table 6.

As seen from Figure 3 and Table 6, the peak time (*T*_max_) of gymnoside I and militarine were 0.16 h and 1.44 h, respectively, both less than 1.5 h, but the *T*_max_ of *α*-isobutylmalic acid was as long as 6.44 h, significantly longer than the *T*_max_ of gymnoside I and militarine. It was shown that the absorption rate of militarine and gymnoside I was faster than that of *α*-isobutylmalic acid *in vivo*. Comparing the mean residence time (MRT) of the three components in the rats, it can be found that the MRT of the three components after intravenous administration was not much different, but the MRT of the three components after oral administration was longer than that after intravenous administration. The results showed that the prolongation of MRT after oral administration mainly came from the influence of gastrointestinal absorption during oral administration, but the extent of MRT prolongation may be due to the transformation relationship between the three components. Related studies have shown that militarine can be rapidly converted into *α*-isobutylmalic acid and gymnoside I *in vivo* [27]. Due to the structural relationship between the three components [21], militarine can be transformed into gymnoside I and *α*-isobutylmalic acid by the action of the gastrointestinal tract *in vivo*. Eventually, the MRT of the three components after oral administration was extended to varying degrees.

At the same time, a double-peak phenomenon was noticed for *α*-isobutylmalic acid (Figure 3). We speculate two possible causes for the same. First, *α*-isobutylmalic acid had an intestinal hepatic circulation in the gastrointestinal tract. Second, due to the metabolic conversion of militarine into *α*-isobutylmalic acid *in vivo*, the continuous supplementation of *α*-isobutylmalic acid in the body led to an increase in the concentration of *α*-isobutylmalic acid in blood, resulting in the bimodal phenomenon.

Although the concentration of militarine in *B. striata* extract was as high as 26.37%, the “absolute bioavailability” of militarine in rats after oral absorption was 0.6%, while *α*-isobutylmalic acid and gymnoside I had better absolute bioavailability *in vivo*, 87.45% and 9.8%, respectively. However, the composition of the extract of traditional Chinese medicine is complicated, and there may be a conversion among the components. The purpose of the concept is to measure the extent of absorption and transformation of militarine, *α*-isobutylmalic acid, and gymnoside I. We observed that militarine had a lower “bioavailability,” indicating that militarine may undergo extensive metabolism *in vivo*. At the same time, due to the structural relationship among militarine, gymnoside I, and *α*-isobutylmalic acid, not only militarine can be converted to *α*-isobutylmalic acid, but also gymnoside I may be converted to *α*-isobutylmalic acid. This indicates that the higher “bioavailability” of *α*-isobutylmalic acid may be due to the conversion of militarine, gymnoside I, and other undetected components so that *α*-isobutylmalic acid has a higher blood concentration *in vivo*. At the same time, the MRT and the area under the curve (AUC) of *α*-isobutylmalic acid and gymnoside I in rats were significantly greater than that of militarine, indicating that the process of therapeutic action of *B. striata in vivo* was likely due to the metabolic conversion of militarine to *α*-isobutylmalic acid and gymnoside I, leading to their higher blood concentrations and longer residence times *in vivo*. However, this speculation needs further experimental verification. The results of this pharmacokinetic and absolute bioavailability study of *B. striata* extract after its oral and intravenous administration in rats could provide the basis for the development of strategies for the clinical application of *B. striata*.

## 4. Conclusion

In this study, the developed LC-MS/MS method for the quantification of *α*-isobutylmalic acid, gymnoside I, and militarine in rat plasma was found to be rapid, sensitive, and reliable. Furthermore, the method was successfully applied to the pharmacokinetic study of *B. striata* extract after intravenous and intragastric administration, and the absolute bioavailability of *α*-isobutylmalic acid, gymnoside I, and militarine was determined to be 87.45%, 9.8%, and 0.6%, respectively. Our results might provide useful information for pharmacokinetic study of other traditional Chinese medicines.

## Figures and Tables

**Table 1:** Precursor/product ion pairs and parameters for MRM of the analytes and the internal standard (IS) puerarin.

Component	Transition (*m/z*)	Cone voltage (V)	Collision energy (eV)
*α*-Isobutylmalic acid	189 ⟶ 129	30	15
Gymnoside I	457.2 ⟶ 285.1	35	15
Militarine	725.3 ⟶ 457.2	40	20
Puerarin (IS)	417.0 ⟶ 267.0	40	30

**Table 2:** Typical equations for calibration curves and lower limit of quantification (LLOQ) (*n* = 3).

Analytes	Calibration curves	Linear range (*μ*g/mL)	*R* ^2^	LLOQ (*μ*g/mL)
*α*-Isobutylmalic acid	*Y* = 0.3148*X* + 0.0072	0.029∼110.33	0.9992	0.029
Gymnoside I	*Y* = 0.333*X* − 0.0002	0.022∼27.50	0.9994	0.022
Militarine	*Y* = 0.2126*X* − 0.0001	0.015∼99.33	0.9991	0.015

**Table 3:** Summary of precision and accuracy of quality control samples added to rat plasma (*n* = 5).

Analytes	Spiked concentration (*μ*g/mL)	Intraday			Interday		
		Calculated concentration (*μ*g/mL)	Precision (RSD%)	Accuracy (%)	Calculated concentration (ng/mL)	Precision (RSD,%)	Accuracy (%)
*α*-Isobutylmalic acid	0.029	0.029 ± 0.001	6.5	101.3	0.028 ± 0.001	5.34	96.6
	4.09	3.84 ± 0.04	10.4	93.9	3.83 ± 0.05	1.3	99.7
	36.78	36.29 ± 1.45	4.0	98.7	36.11 ± 1.21	3.4	98.2

Gymnoside I	0.02	0.02 ± 0.001	4.6	100.9	0.02 ± 0.001	5.5	100.4
	2.75	2.62 ± 0.09	3.3	95.4	2.60 ± 0.09	3.5	94.5
	13.75	13.19 ± 0.02	1.5	95.9	13.45 ± 0.37	2.8	97.8

Militarine	0.015	0.014 ± 0.002	11.4	93.3	0.014 ± 0.001	8.4	93.3
	2.64	2.57 ± 0.05	1.9	97.3	2.60 ± 0.06	2.3	98.5
	23.78	23.03 ± 1.34	5.8	96.8	23.29 ± 1.13	4.9	97.9

**Table 4:** Matrix effects and extraction recoveries of the three compounds (*n* = 5).

Analytes	Spiked concentration (ng/mL)	Extraction recovery		Matrix effect	
		Mean ± SD (%)	RSD (%)	Mean ± SD (%)	RSD (%)
*α*-Isobutylmalic acid	0.029	99.68 ± 4.50	4.5	93.21 ± 3.54	3.8
	4.09	95.77 ± 1.12	1.2	96.45 ± 0.78	0.8
	36.78	100.98 ± 2.14	2.1	100.09 ± 2.45	2.4

Gymnoside I	0.22	93.99 ± 6.47	6.9	98.73 ± 7.59	7.7
	2.75	92.65 ± 8.09	8.7	101.74 ± 3.34	3.3
	13.75	94.71 ± 4.03	4.3	99.08 ± 0.67	0.7

Militarine	0.015	100.16 ± 8.81	8.8	94.81 ± 1.56	1.6
	2.64	94.78 ± 2.17	2.3	95.63 ± 2.53	2.6
	23.78	99.66 ± 5.36	5.4	99.91 ± 6.36	6.4

**Table 5:** Summary of stability of quality control samples added to rat plasma (*n* = 5).

Analytes concentration (*μ*g/mL)	Spiked concentration (*μ*g/mL)	12 h, 4°C			3 freeze-thaw cycles			6 h, room temperature		
		Calculated concentration (*μ*g/mL)	Precision (RSD, %)	Accuracy (%)	Calculated concentration (*μ*g/mL)	Precision (RSD, %)	Accuracy (%)	Calculated concentration (*μ*g/mL)	Precision (RSD, %)	Accuracy (%)
*α*-Isobutylmalic acid	0.029	0.028 ± 0.002	7.1	96.6	0.027 ± 0.001	3.7	93.1	0.03 ± 0.001	3.3	103.4
	4.09	3.92 ± 0.11	2.8	95.8	3.83 ± 0.08	10.4	93.6	3.77 ± 0.09	2.4	92.2
	36.78	34.71 ± 1.95	6.1	94.4	34.17 ± 1.28	3.7	92.9	34.95 ± 1.89	5.4	95.0

Gymnoside I	0.022	0.021 ± 0.001	4.8	95.4	0.022 ± 0.001	4.8	100.3	0.022 ± 0.001	4.5	100.2
	2.75	2.68 ± 0.15	6.0	97.5	2.74 ± 0.10	3.6	99.6	2.77 ± 0.08	3.6	99.6
	13.75	13.22 ± 0.49	3.7	96.1	13.92 ± 0.57	5.2	101.2	13.69 ± 0.39	5.2	101.2

Militarine	0.015	0.015 ± 0.001	6.7	100.1	0.015 ± 0.001	6.7	100.7	0.016 ± 0.001	6.2	106.7
	2.64	2.46 ± 0.28	11.4	93.2	2.62 ± 0.09	3.4	99.2	2.62 ± 0.09	3.4	99.2
	23.78	21.95 ± 1.1	5.0	92.3	21.8 ± 0.94	4.3	91.7	21.3 ± 0.94	4.4	89.6

**Table 6:** Pharmacokinetic parameters of the three analytes after oral and intravenous administration of *B. striata* extract to rats (*n* = 6).

Parameters	Unit	Intravenous			Oral		
		*α*-Isobutylmalic acid	Gymnoside I	Militarine	*α*-Isobutylmalic acid	Gymnoside I	Militarine
*T*_max_	*h*	—	—	—	6.44 ± 2.33	1.44 ± 2.29	0.16 ± 0.11
*C*_max_	*μ*g/mL	—	—	—	23.18 ± 5.42	3.06 ± 4.82	3.30 ± 3.60
AUC_0–*t*_	*h∗μ*g/mL	38.7 ± 4.72	15.47 ± 1.63	36.71 ± 5.66	458.12 ± 150.74	20.56 ± 12.05	3.10 ± 1.16
AUC_0–∞_	*h∗μ*g/mL	39.62 ± 4.26	17.52 ± 2.66	37.22 ± 5.99	498.36 ± 173.09	21.81 ± 12.04	4.26 ± 1.93
MRT_0–*t*_	*h*	0.45 ± 0.08	0.68 ± 0.07	0.36 ± 0.06	15.59 ± 3.63	12.42 ± 3.73	5.62 ± 2.45
MRT_0–∞_	*h*	0.51 ± 0.1	0.93 ± 0.18	0.39 ± 0.07	19.69 ± 6.25	15.98 ± 5.34	9.31 ± 2.57
Bioavailability	%	—	—	—	87.4%	9.8%	0.6%

## Data Availability

The data used to support the findings of this study are included within the article.
